# Reactivation-Dependent Amnesia for Contextual Fear Memories: Evidence for Publication Bias

**DOI:** 10.1523/ENEURO.0108-20.2020

**Published:** 2021-01-06

**Authors:** Natalie Schroyens, Eric L. Sigwald, Wim Van Den Noortgate, Tom Beckers, Laura Luyten

**Affiliations:** 1 Centre for the Psychology of Learning and Experimental Psychopathology, Faculty of Psychology and Educational Sciences, Katholieke Universiteit Leuven, Leuven 3000, Belgium; 2 Leuven Brain Institute, Leuven 3000, Belgium; 3 Laboratorio de Neuropatología Experimental, Instituto de Investigación Médica Mercedes y Martín Ferreyra-Consejo Nacional de Investigaciones Científicas y Técnicas-Universidad Nacional de Córdoba, Córdoba 5016, Argentina; 4 Faculty of Psychology and Educational Sciences and ITEC (interdisciplinary research group of imec and Katholieke Universiteit Leuven), Katholieke Universiteit Leuven, Kortrijk 8500, Belgium; 5 Methodology of Educational Research, Faculty of Psychology and Educational Sciences, Katholieke Universiteit Leuven, Leuven 3000, Belgium

**Keywords:** amnesia, contextual fear memory, pharmacology, publication bias, reconsolidation, rodents

## Abstract

Research on memory reconsolidation has been booming in the last two decades, with numerous high-impact publications reporting promising amnestic interventions in rodents and humans. However, our own recently-published failed replication attempts of reactivation-dependent amnesia for fear memories in rats suggest that such amnestic effects are not always readily found and that they depend on subtle and possibly uncontrollable parameters. The discrepancy between our observations and published studies in rodents suggests that the literature in this field might be biased. The aim of the current study was to gauge the presence of publication bias in a well-delineated part of the reconsolidation literature. To this end, we performed a systematic review of the literature on reactivation-dependent amnesia for contextual fear memories in rodents, followed by a statistical assessment of publication bias in this sample. In addition, relevant researchers were contacted for unpublished results, which were included in the current analyses. The obtained results support the presence of publication bias, suggesting that the literature provides an overly optimistic overall estimate of the size and reproducibility of amnestic effects. Reactivation-dependent amnesia for contextual fear memories in rodents is thus less robust than what is projected by the literature. The moderate success of clinical studies may be in line with this conclusion, rather than reflecting translational issues. For the field to evolve, replication and non-biased publication of obtained results are essential. A set of tools that can create opportunities to increase transparency, reproducibility and credibility of research findings is provided.

## Significance Statement

The present study suggests that the literature on drug-induced, reactivation-dependent amnesia for contextual fear memories in rodents is biased. Such bias is problematic because it can misinform researchers when making decisions on how to optimally invest their resources. The lack of robustness of amnestic effects, in combination with the strict (but vague) conditions that are required for memory destabilization and the absence of clear explanations for some of the observed null effects, casts doubt on the potential of the proposed clinical application of postreactivation interventions. Finally, current mechanistic theories that are commonly used to explain reactivation-dependent amnesia, such as reconsolidation or state dependency, cannot predict when amnestic effects will occur.

## Introduction

Amnesia for previously acquired memories can be obtained by applying certain treatments shortly before or after memory reactivation. After the first published observation of reactivation-dependent amnesia, which was obtained by giving rats an electroconvulsive shock after a brief, unreinforced re-exposure to a conditioned tone (Misanin et al., 1968), this procedure was conceptually replicated using a wide variety of experimental protocols and treatments in different species (Reichelt and Lee, 2013; Beckers and Kindt, 2017). Overall, research on reactivation-dependent amnesia, commonly referred to as “reconsolidation blockade,” has accelerated during the last two decades, with high-impact publications reporting promising amnestic interventions in rodents and humans (Nader et al., 2000; Kindt et al., 2009).

Meanwhile, studies revealed that memory reactivation does not occur each time a memory is retrieved but depends on the conditions under which the memory was acquired and retrieved (e.g., memory strength, age, type, or the amount of novelty introduced during the reactivation session; Tronson and Taylor, 2007). Apart from those controlled studies that examined limiting factors on memory destabilization, there have been only few papers reporting failures to obtain reactivation-dependent amnesia under standard conditions. Published failures mainly involved pharmacologically-induced amnesia in human participants (Bos et al., 2014; Thome et al., 2016; Schroyens et al., 2017) or the retrieval-extinction effect (Soeter and Kindt, 2011; Luyten and Beckers, 2017; Chalkia et al., 2020a), whereas the literature on pharmacologically-induced amnesia for fear memories in rodents shows robust and consistent amnestic effects, with hardly any failures to replicate.

In our lab, we aimed to further investigate opportunities for the clinical application of reactivation-dependent amnesia for fear memories. To this end, we set out to replicate published studies in which systemic drug injection after unreinforced re-exposure to a conditioned stimulus (CS) in rats resulted in amnesia for contextual or cued fear memories (Schroyens et al., 2019a,b; Luyten et al., 2020). In contrast to what is reported in the literature, our extensive series of conceptual and exact replication attempts, performed by several experimenters and in different laboratories, did not provide clear evidence for reactivation-dependent amnesia. In fact, we could only reproduce the amnestic effect when the experimenter from the original study (now a member of our research group) conducted the study in the original lab, with animals purchased from the local supplier [see Table 1 for an overview of our exact replication attempts using contextual fear conditioning and midazolam (MDZ)]. Given that we tested a wide range of behavioral parameters and (sometimes exactly) adhered to the standard protocols that have been typically used in the literature, it is unlikely that our negative results can be explained by previously established limiting factors on memory destabilization.

**Table 1:** Overview of our exact replication attempts using contextual fear conditioning and postreactivation systemic MDZ injection in male Wistar rats

Experiment	Correspondence between original and replication study		Amnestic effect observed? (MDZ < SAL)	Sample size	Obtained power
	Researcher	Lab space			
JA09^1^	x		No	12	0.87
JA10^1^	x		No	15	0.93
JA11^1^	x	x	Yes	20	0.98
JA12^1^	x	x	Yes	19	0.97
NS09^1^			No	12	0.87
NS11^2^			No	12	0.68
NSARG01^3^		x	No	16	0.99

In these experiments, the methodology of the original studies was followed as closely as possible. All studies involved contextual fear conditioning, followed one day later by brief (i.e., 2, 3, or 5 min) unreinforced re-exposure to the conditioned context and systemic injection of MDZ (1.5 or 3 mg/kg) or saline (SAL), and retention testing one day later.

^1^Exact replication of Alfei et al. (2015) and Ferrer Monti et al. (2017).

^2^Exact replication of Stern et al. (2012).

^3^Exact replication of Espejo et al. (2016) and Ortiz et al. (2015).

The table indicates whether the experimenter and the lab in which the replication study was performed were the same as in the published, original study. The results show that the amnestic effect could not be replicated when the study was performed in a different lab or by another researcher, despite adherence to the experimental protocol of the original studies. These findings illustrate that the success of treatment may depend on subtle between-study differences, and the underlying causes of these failures to replicate remain unknown. Obtained power for our sample sizes, shown in the last column, was calculated using the smallest effect size (Hedges’ *g*) that was observed in the original studies (α = 0.05; *d* = 1.71 in Alfei et al., 2015; 1.31 in Stern et al., 2012; 2.91 in Espejo et al., 2016; see Table 2). A complete description of our replication attempts involving contextual fear can be found in Schroyens et al. (2019a,b). Appendix A (Tables A.1 and A.2) of Schroyens et al. (2019a) contains a detailed overview of experimental parameters for the conceptual and exact replication attempts, respectively. Experiment NSARG01 is described in Schroyens et al. (2019b).

Overall, it can be concluded that the experimental evidence obtained in our replication attempts is not in line with the general representation of amnesia by postreactivation systemic drug injection in the literature. Our five failed exact replication attempts using contextual fear and MDZ (Table 1) suggest that the outcome of the procedure depends on delicate, unknown, and possibly uncontrollable parameters. Therefore, it seems unlikely that the high rate of large amnestic effects that is portrayed by the current literature is a reliable representation of actual observations. Based on the discrepancy between our results and those from published studies, we suspect that (1) amnestic effects are less easily replicated than what is currently suggested by the literature and thus (2) the large effect sizes that are reported in the literature are merely a subset of the range of effect sizes that have effectively been observed. We hypothesize that these issues arise from the omission of negative findings in the published literature (i.e., reporting and publication bias).

The main aim of the current paper was to assess whether indeed the literature on pharmacologically-induced reactivation-dependent amnesia for contextual fear memories in rodents shows evidence of publication bias. The first part of this project, which we completed before preregistration, consisted of an exploratory assessment of publication bias in the sample of published studies that used postreactivation systemic injection of MDZ. Given that our ultimate aim was to investigate whether publication bias applies to the field in a broader sense, rather than for just one (systemically injected) drug, we performed a preregistered systematic review of the literature on pharmacologically-induced reactivation-dependent amnesia for contextual fear memories in rodents. Publication bias in this larger sample was assessed statistically, and relevant researchers were contacted to enquire about and request unpublished datasets. The obtained results contribute to a clearer view on the robustness of reactivation-dependent amnesia for contextual fear memories in rodents.

## Materials and Methods

Relevant datasets, R scripts and overview tables of all included studies can be found on the Open Science Framework (OSF) at https://osf.io/apu9t/ (DOI 10.17 605/OSF.IO/APU9T).

### Systematic literature review

We performed a literature search through the online database of PubMed using the Boolean search terms ‘(context OR contextual) AND (fear OR aversive OR threat) AND (memory OR learning) AND (reconsolid* OR reactivat* OR destabili*)’ to look for relevant published papers concerning drug-induced reactivation-dependent amnesia for contextual fear memories in rodents. After obtaining in-principle acceptance for the present study, the systematic review was registered at PROSPERO, in which we further specified that “pharmacological manipulations” do not entail genetic manipulations and, given that we investigate “reactivation-dependent amnesia,” we only consider treatments that were aimed at inducing amnesia (these criteria were implied, but not explicitly mentioned, in the Stage 1 Registered Report).

### Inclusion criteria

Experiments were included when meeting all of the following criteria (related to each element of the PICO framework):

(1) Population. Rats or mice of either sex were used.

(2) Intervention. Contextual fear conditioning [i.e., one or multiple unsignaled shock(s) administered in the training context] and, afterward, a pharmacological manipulation was applied once before or after a brief unreinforced re-exposure to the training context that is commonly referred to as “contextual fear memory reactivation.” Experiments were included regardless of the mode of drug administration.

(3) Control group. A negative control group was included, in which subjects received a memory reactivation session combined with vehicle administration, or in which the drug of interest was administered without receiving a memory reactivation session. If multiple negative control groups were used, the most-commonly used control was considered, which appeared to be the vehicle control. Experiments that did not include such a control group, but, for example, only a positive control condition (i.e., in which the treatment of interest is compared with a “gold standard” treatment) were not included in the meta-analyses because of a lack of appropriate control.

(4) Outcomes. A behavioral measure of fear or anxiety (e.g., freezing) was included during drug-free testing for long-term memory retention (at least one day after reactivation). If multiple tests were performed, only the results of the first drug-free long-term retention test were included.

(5) Studies for which we were unable to calculate the effect size from reported graphs or statistics are addressed in the paper but not included in the meta-analyses.

We excluded from the meta-analysis those “boundary” conditions in which amnesia is not expected to occur based on theoretical considerations and prior empirical observations concerning reactivation-dependent amnesia. As mentioned in the introduction, it is established that reactivation-dependent amnesia occurs only under certain theoretically-grounded circumstances. For example, it has been found that the success of obtaining reactivation-dependent amnesia depends on memory-related characteristics (such as its age or strength), the use of (stressful) interventions before learning, the conditions under which memory is retrieved (e.g., properties and duration of the reactivation session), the timing of drug application (e.g., not too long before/after the reactivation session), and the time of retention testing (e.g., amnesia is not expected to be observed immediately after the intervention). Importantly, we did not aim to investigate the presence of null findings obtained under those boundary conditions. Rather, we wanted to assess whether negative results have been obtained (and possibly suppressed) in situations where amnesia was expected to occur (i.e., under standard conditions). However, given that these limiting conditions are not absolute (i.e., they can be overcome and seem to interact with each other), it is impossible to predefine a comprehensive set of exact conditions in which amnesia is (not) expected to occur. Therefore, the experimental parameters of all experiments that fulfill the criteria stated above (see ‘Inclusion criteria’) were summarized in an overview table and reviewed independently by two other researchers to select relevant studies to be included in the meta-analyses. Both researchers have experience in the topic, were blinded for study outcome, and judged inclusion based on the guiding principles listed below. Given the widely accepted boundary conditions for fear memory destabilization, memories should be recent (<7 d) at the time of reactivation, and the reactivation session should take less than two times the duration of the training session. For studies that explicitly aimed to investigate conditions that were expected to impede reactivation-dependent amnesia (such as, for example, stress manipulations before learning), only the “positive control” condition (in which the effect was expected to occur) are included, whereas the conditions under investigation are excluded (regardless of their outcome, given that the selecting researchers were blinded). Negative control conditions that are commonly used in the investigation of amnesia, such as delayed treatment application (>1 h after termination of the reactivation session) or short-term memory tests, were excluded from the meta-analysis. Nevertheless, as mentioned earlier, any conditions that met the first four inclusion criteria listed in the previous paragraph were included in an overview table (https://osf.io/x2pkq/). This way, a thorough overview of all adopted experimental parameters and boundary conditions is provided.

Articles were selected based on the abstract, and the methods section was screened as well if the abstract provided no information on the inclusion of a contextual fear memory procedure and/or insufficient information regarding drug application. Afterwards, the full text of the selected articles was screened to further assess eligibility (see https://osf.io/qebtd/ for a detailed overview of the review process). The summary table providing detailed information on experimental parameters for all included studies can be found at https://osf.io/sjwbd/. Based on this information, two blinded researchers further selected conditions for inclusion in the meta-analysis. For the purpose of restricting the amount of papers to be included in the meta-analysis and the number of researchers to be contacted (see ‘Acquiring unpublished data’ below), we limited the scope of the meta-analysis to the most commonly-used drugs to induce reactivation-dependent amnesia for contextual fear memories. Therefore, only studies that met all above-mentioned inclusion criteria (see ‘Inclusion criteria’) and used drugs that appeared in five or more research articles [i.e., anisomycin (ANI), MDZ, MK-801, and propranolol (PROP)] were included in the meta-analyses.

### Calculation of Hedges’ *g*

Means and SDs were estimated from reported descriptive and test statistics or from reported graphs using WebPlotDigitizer (Rohatgi, 2019). If only the overall sample size of a study was provided and the group sizes could not be derived, we assumed that subjects were equally divided among the groups. Hedges’ *g* (with correction for small-sample bias) and corresponding SE were calculated based on our estimates from means, SDs, and group sizes using the metafor package in R. The Stage 1 version of the Registered Report mentioned “Cohen’s *d*” instead of “Hedges’ *g*.” However, we decided to use Hedges’ *g* (which is the default output of the adopted escalc function of the metafor package when calculating standardized mean differences), because this measure corrects for small-sample bias. In any case, we did compare both measures of effect sizes for all included studies and found highly similar estimates.

### Meta-analysis

We used the metafor package in R to fit meta-analytic random-effects models, using restricted maximum likelihood (Viechtbauer, 2010). Measures of between-study variation include τ^2^, I^2^, and Cochran’s Q test. Research group and amnestic drug were included as moderators in case of significant between-study heterogeneity. Importantly, rather than estimating the size of the amnestic effect or investigating moderators, our goal was to assess whether the overall sample of published studies is subject to publication bias.

### Publication bias

The funnel plot, in which the effect estimate for each study (here, the standardized mean difference) is plotted against a measure of precision of that study (here, the SE of the standardized mean difference as suggested by Sterne and Egger, 2001), is a primary visual tool to assess publication and other biases (Sterne et al., 2005; Peters et al., 2008). Observed effect sizes such as standardized mean differences are unbiased estimates of the population effect size regardless of the sample size, but the effect sizes obtained by studies with relatively small precision are in general more variable than those from studies with higher precision. As a result, in the absence of bias, those small-precision studies (i.e., lying at the bottom of the plot) are expected to scatter more widely compared with large-precision studies (lying at the top of the plot), resulting in a symmetrical funnel-shape of the dots in the plot. However, if small studies with non-significant results remain unreported or unpublished, we can expect a gap (located at the left bottom side in case of a positive true effect size) and the funnel shape can thus become asymmetrical. Egger’s linear regression approach was used to assess such plot asymmetry. We used a weighted regression of the effect estimates on their SEs, including a multiplicative dispersion parameter (Sterne and Egger, 2005).

Although funnel plots and Egger’s regression are standard tools for the assessment of publication bias, it should be noted that publication bias is not the only possible cause of funnel plot asymmetry or a relationship between study precision and effect size (Sterne et al., 2005). For example, between-study heterogeneity in itself may lead to funnel plot asymmetry because of an accidental correlation between precision and effect size or because of a confounding effect of study characteristics. Such heterogeneity can pose a challenge for funnel plot interpretation. Consider, for example, the research group in which the experiments were performed: certain environmental or methodological differences between research groups may lead to differences in both observed effect sizes and precision of studies (researchers that obtain large effects might evolve to using smaller samples in their future studies). In order to account for such heterogeneity in observed effect sizes, research group was included as a moderator. The meta-analytic model without moderators was used for the creation of the funnel plots (which allowed for plotting of the raw effect sizes rather than their residual values), whereas the model with moderators was used for Egger’s linear regression (allowing to statistically test for funnel plot asymmetry after accounting for the influence of the moderator(s) included in the meta-analytic model). For the sake of completeness, results of all regression models (with and without moderators) can be found at https://osf.io/zshwx/.

Apart from publication bias and genuine between-study heterogeneity, other sources of reporting bias (e.g., selective outcome or analysis reporting), suboptimal design and/or analyses used in smaller studies, and artefactual sampling variance may also lead to non-asymmetric funnel plots (Sterne et al., 2011). One way to discriminate publication bias as a source of asymmetry in funnel plots from other factors is by using contour-enhanced funnel plots (Peters et al., 2008). Contour-enhanced funnel plots, in which levels of statistical significance are displayed (i.e., <0.01, <0.05, and <0.1), were therefore used to visualize whether publication bias is a likely factor contributing to funnel plot asymmetry (Peters et al., 2008).

### Acquiring unpublished data

All corresponding authors from the selected articles and other relevant researchers were contacted via E-mail to enquire about and request unpublished datasets. In addition, announcements were spread using StudySwap (Chartier et al., 2018), conference mailing lists, and social media (Twitter, ResearchGate, etc.). Obtained unpublished datasets that met the inclusion criteria stated above (see ‘Inclusion criteria’) were included in funnel plots to get an indication of the precision, obtained effect sizes, and statistical significance of these unpublished results. In addition, Egger’s regression was repeated using the total sample that includes published as well as unpublished datasets.

### Pilot data

Before preregistration of the current study, we completed some exploratory analyses. In the course of reporting some of our replication efforts (Schroyens et al., 2019a), we performed a thorough literature search for studies that, like in our experiments, had used contextual fear conditioning and postreactivation systemic injection of MDZ to induce amnesia for a previously acquired fear memory in adult rats. We found 15 published papers (until April 2019; see Table 2) and conducted a random-effects meta-analysis using this sample (adhering to the inclusion criteria and statistical analyses outlined in the methods section above). The same analyses were also performed on datasets from our own replication studies (Schroyens et al., 2019a,b), in which highly similar procedures and parameters were used.

**Table 2:** Experiments (until April 2019) from 15 different papers included in our pilot analyses investigating amnestic effects of postreactivation MDZ administration for contextual fear conditioning in adult rats under standard conditions (in chronological order based on publication date)

Publication	Research group	Exp.	Figure	Reactivation session duration	MDZ dose (mg/kg)	*N*_total_	Effect size (Hedges’ *g*)
Bustos et al. (2006)	I	1B	2*B*	90 s	1	16	1.93*
Bustos et al. (2006)	I	2A	3*B*	90 s	1	17	2.40*
Bustos et al. (2006)	I	2A	3*B*	90 s	1	17	2.34*
Bustos et al. (2006)	I	3B	7*B*	90 s	1	16	4.60*
Zhang and Cranney (2008)	II	1	1	90 s	2	18	0.96*
Zhang and Cranney (2008)	II	3	3	90 s	2	16	1.76*
Bustos et al. (2009)	I	NA	1*B*	1 min	1.5	14	0.74
Bustos et al. (2009)	I	NA	1*C*	3 min	1.5	14	3.63*
Bustos et al. (2009)	I	NA	1*D*	5 min	1.5	20	2.95*
Bustos et al. (2009)	I	NA	2*C*	10 min	1.5	15	−2.06
Bustos et al. (2009)	I	NA	3*B*	3 min	1.5	14	3.13*
Bustos et al. (2010)	I	1	1*B*	3 min	1.5 or 3	17	2.52*
Bustos et al. (2010)	I	1	1*C*	5 min	1.5 or 3	19	2.41*
Stern et al. (2012)	III	1	1*A*	3 min	1.5	20	1.31*
Pineyro et al. (2013)	IV	1	1*C*	1 min	3	12	0.04
Pineyro et al. (2013)	IV	1	1*C*	4 min	3	12	3.53*
Pineyro et al. (2013)	IV	1	1*C*	5 min	3	12	2.96*
Alfei et al. (2015)	IV	5	5*A*	2 min	3	18	1.71*
Alfei et al. (2015)	IV	6	6*B*	5 min	3	14	2.59*
Ortiz et al. (2015)	I	1	1*C*	3 min	3	19	3.22*
Ortiz et al. (2015)	I	1	1*E*	5 min	3	12	4.11*
Ortiz et al. (2015)	I	2	3*C*	5 min	3	16	4.10*
Ferrer Monti et al. (2016)	IV	1	1*C*	90 s	3	15	–0.05
Ferrer Monti et al. (2016)	IV	1	1*C*	4 min	3	14	2.79*
Espejo et al. (2016)	I	1	1	5 min	3	15	3.92*
Espejo et al. (2016)	I	2	2	5 min	3	22	2.91*
Saitoh et al. (2017)	V	NA	2*B*	3 min	1	24	0.85*
Ferrer Monti et al. (2017)	IV	2	2*B*	2 min	3	12	3.58*
Espejo et al. (2017)	I	1	1	5 min	3	16	3.30*
Espejo et al. (2017)	I	3	3	5 min	3	18	3.73*
Akagi et al. (2018)	V	NA	2*C*	3 min	1	30	0.83*
Franzen et al. (2019)	III	NA	1*A*	1 min	3	18	–0.12
Franzen et al. (2019)	III	NA	1*B*	2 min	3	17	1.39*

Adapted from Schroyens et al. (2019a). See Inclusion criteria for an overview of which conditions were included. Studies that used contextual fear conditioning and postreactivation systemic MDZ injection in rats were included after a thorough literature search. Note that two additional papers with MDZ studies were identified by our systematic PubMed search and included in the preregistered analyses (De Oliveira Alvares et al., 2013; Couto-Pereira et al., 2019). The effect size (Hedges’ *g*) for the influence of MDZ on % freezing during the test session was estimated based on means and SEs from reported graphs (MDZ vs vehicle) using the metafor package in R. Details of the intervention, such as duration of the training and reactivation session and drug dose, are indicated as well.

*Amnestic effects reported as significant (at an α level of 0.05). The numbers in the second column refer to the research group,

I, IFEC-CONICET, Departamento de Farmacología, Facultad de Ciencias Químicas, Universidad Nacional de Córdoba, Córdoba, Argentina.

II, School of Psychology, University of New South Wales, Sydney, Australia.

III, Department of Pharmacology, Federal University of Santa Catarina, Florianópolis, Santa Catarina, Brazil.

IV, Laboratorio de Psicología Experimental, Facultad de Psicología, Universidad Nacional de Córdoba, Córdoba, Argentina.

V, Department of Neuropsychopharmacology, National Institute of Mental Health, National left of Neurology and Psychiatry, Tokyo, Japan.

Each of the 15 published papers contained at least one study in which an amnestic effect was found. Some papers included conditions that aimed to test limiting factors on reactivation-dependent amnesia, i.e., (stressful) interventions before learning (Zhang and Cranney, 2008; Bustos et al., 2010; Ortiz et al., 2015; Espejo et al., 2016, 2017), the use of remote fear memories (Bustos et al., 2009), reactivation durations that yield inadequate levels of prediction error (Alfei et al., 2015), or drug injection outside the reconsolidation time window (Bustos et al., 2006; Stern et al., 2012). Based on the inclusion criteria described in the methods section, those conditions in which amnestic effects were not hypothesized to occur, were not included in the present meta-analysis given that we aimed to study the occurrence of reactivation-dependent amnesia under optimal standard conditions. Included studies in which no amnestic effects were found used either relatively brief (i.e., 1 or 1.5 min) or long (i.e., 10 min) reactivation sessions. A complete overview of experimental parameters adopted in each of the studies can be found on our OSF page at https://osf.io/sjwbd/. If multiple intervention groups were compared with the same control group, the intervention groups were combined into a single group as recommended by Higgins and Green (2011).

### Published studies from other research groups versus our own replication attempts using MDZ: a first indication of publication bias

**Figure 1.**
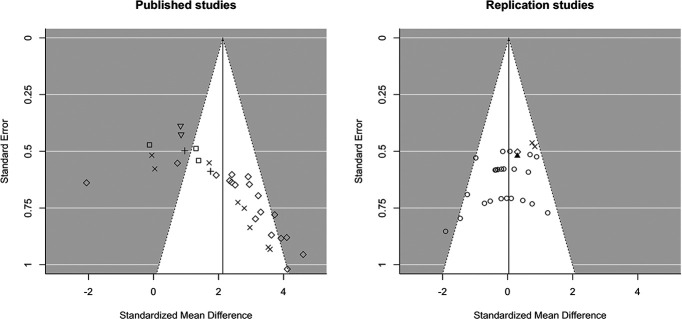
Funnel plots including published studies (left panel) and our own replication studies (right panel) in which MDZ was used as amnestic agent. Each point represents an observed effect size Hedges’ *g* against its SE. Visual inspection of the plot on the right panel shows that our replication studies are symmetrically scattered around the effect estimate of 0.04, indicating that the estimated effect size is close to zero and suggesting that no trend in one particular direction was observed across studies. In contrast, the plot of published studies (left panel) clearly shows asymmetry, and the reported effect sizes seem to depend strongly on the research group in which the studies were performed (represented by the different symbols in the left plot). Egger’s test confirmed plot asymmetry (*p* < 0.0001), even when considering the moderating influence of research group. One should be careful to attach value to the estimated effect size shown in the left funnel plot, given the evidence for publication bias and because the nesting of studies within research groups is not accounted for. The funnel plots were based on the meta-analytic models without moderators. Symbols represent the research group in which each study was performed (left panel) or the lab space that was used (right panel). Note that three of our exact replication studies (right panel) were performed in the same lab space as some of the original, published studies (left panel).

An extensive literature search for studies using contextual fear conditioning and systemic MDZ injection after memory reactivation revealed 15 papers, containing a total of 33 comparisons (postreactivation MDZ vs SAL) that fulfilled the standard conditions for memory destabilization (Table 2). Visual inspection of the funnel plot including these experiments suggests asymmetry (Fig. 1, left panel). The random-effects meta-analysis on this sample (k = 33, total *N* = 549) showed considerable between-study heterogeneity [Q(32) = 164.10; *p* < 0.001; τ^2^ = 1.76 (SE = 0.55) [0.98; 3.45]; I^2^ = 81.84% [71.45; 89.83]], implying differences between studies beyond those to be expected by chance. Given such heterogeneity, and as preregistered, research group was included in the model as a moderator. We found that the effect sizes plotted in Figure 1, left panel, depended on the research group in which the experiment was performed [i.e., research group was a statistically significant moderator of effect size; QM(5) = 82.35, *p* < 0.001]. Nevertheless, residual heterogeneity remained significant [QE(28) = 124.78; *p* < 0.001; τ^2^ = 1.53 (SE = 0.53) [0.78; 3.15]; I^2^ = 78.40% [64.92; 88.24]], suggesting that reported effect sizes differ significantly between research groups, but these between-group differences cannot fully explain all of the observed heterogeneity between studies. When statistically assessing the relationship between the effect estimates and their SEs (i.e., the relation represented in the funnel plots), we used the meta-analytic model with the moderator to test for funnel plot asymmetry after accounting for the influence of research group. Doing so, Egger’s test provided statistical evidence for funnel plot asymmetry (*t*_(27)_ = 5.02; *p* < 0.001), which can be an indication of publication bias.

A similar random-effects meta-analysis on the replication studies from our group (k = 27, *N* = 324) showed different results (Fig. 1, right panel). No signficant between-study heterogeneity was observed [Q(26) = 34.13; *p* = 0.132; τ^2^ = 0.08 (SE = 0.12) [0.00; 0.60]; I^2^ = 19.34% [0.00; 62.95]]. Visual inspection of this plot shows a different pattern than the one obtained for previously published studies, as our studies seem to be scattered more symetrically. Nevertheless, Egger’s regression test indicated a significant negative relationship between the effect estimates and their SEs (*t*_(25)_ = −2.46; *p* = 0.021), possibly because of the use of a small sample size of 4 rats/group in a few of our studies (i.e., those represented on the bottom left of the graph) providing inaccurate effect estimates.

**Figure 2.**
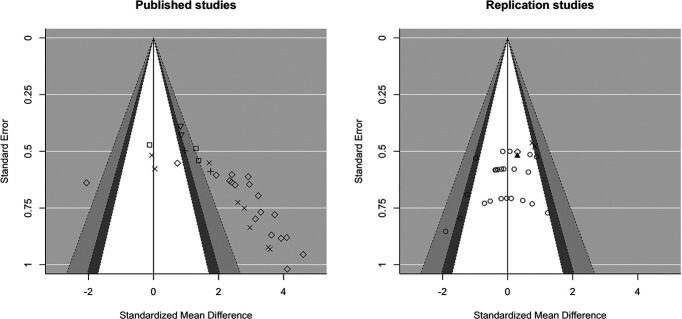
The contour-enhanced funnel plot of published studies (left panel) suggests publication bias. Published studies (left panel) are missing in the white area and the region to the left of the white area (where non-significant results would be plotted). This pattern adds credibility to the possibility that funnel plot asymmetry is caused by publication bias based on statistical significance (Peters et al., 2008). As a comparison, our own (mainly non-significant) replication studies are plotted in the right graph. Symbols represent the research group in which each study was performed (left panel) or the lab space that was used (right panel). The white area and the region to the left of the white area contain non-significant one-sided *p* values (white region: *p* values between 0.05 and 0.95; dark gray-shaded region: *p* values between 0.95 and 0.975; medium gray-shaded region: *p* values between 0.975 and 0.995, light gray-shaded region outside of the funnel: *p* values > 0.995); areas to the right of the white area represent statistically significant one-sided *p* values (dark gray-shaded region: *p* values between 0.025 and 0.05; medium gray-shaded region: *p* values between 0.005 and 0.025, light gray-shaded region outside of the funnel: *p* values below 0.005).

The funnel plot and Egger’s test thus clearly reveal asymmetry in the published studies, which might indicate publication bias. One way to discriminate publication bias as a source of asymmetry in funnel plots from other factors is by using contour-enhanced funnel plots (Peters et al., 2008). The contour-enhanced funnel plot indicates that nearly all published studies report significant results (i.e., studies plotted to the right of the white area are statistically significant in a one-tailed test; Fig. 2, left). The fact that studies seem to be missing in the white area of the plot suggests that suppression of non-significant results is likely a factor contributing to funnel plot asymmetry. Again, this plot is in stark contrast to the one displaying our replication studies, in which most studies yielded non-significant results (Fig. 2, right).

### Published studies from other research groups versus our replication attempts using MDZ: the subtle nature of reactivation-dependent amnesia

Comparing both funnel plots (Fig. 1) not only suggests publication bias, but also illustrates that the effect sizes obtained in our studies were never as large as in published research. As mentioned earlier, we hardly found statistical evidence for the presence of (large) amnestic effects, while published studies show quite the opposite pattern; they suggest that negative results are rarely obtained in this field (Fig. 2). This highlights that, even in our exact replication attempts, there were inherent differences between our own and published studies that determined the success of the intervention. In line with this observation, the meta-analysis on the sample of published studies showed that the research group in which experiments were performed significantly affected the obtained effect size (see previous paragraph). Such dependence highlights the subtle nature of reactivation-dependent amnesia and raises the question whether other research groups also conducted unsuccessful attempts to obtain amnestic effect (but never published those findings). The results of our preregistered analyses (see results section below) address this question in depth and provide more insight into the overall robustness of postreactivation amnesia for contextual fear memories in rodents.

## Results

### Funnel plots and Egger’s regression suggest publication bias

The preregistered systematic PubMed search identified 304 articles, 89 of which met our inclusion criteria. The wide range of drugs that has been used with the purpose of inducing reactivation-dependent amnesia for contextual fear memories in rodents is shown in Table 3 (systemic administration) and Table 4 (intracranial administration). The scope of the meta-analysis was narrowed down to reactivation-dependent amnesia induction under standard conditions and with commonly-used amnestic drugs (i.e., those that appeared in five or more of the identified research articles, which were found to be ANI, MDZ, PROP, and MK-801). “Standard” conditions were defined based on theoretical considerations in line with the fear memory reconsolidation account (see Inclusion criteria). The final sample that was included in the preregistered meta-analysis consisted of 52 research articles, containing a total of 77 experiments and 95 drug-vehicle comparisons. It should be noted that one of those papers, i.e., Espejo et al. (2016), was not identified via the systematic PubMed search, but given that the study was already included in the pilot study, we also included it in the present analyses. In addition, we did not include Schroyens et al. (2009a,b; those were included in separate analyses; see Figs. 1, 2, right panels) as we aimed to review the literature that originated from outside our own research group. A detailed overview of experimental parameters used in the studies that were included in the meta-analyses can be found at https://osf.io/sjwbd/.

**Table 3:** Overview of studies in which pharmacological agents were administered systemically with the aim of inducing reactivation-dependent amnesia for contextual fear memories in rodents, as identified by the systematic review

Target/function	Drug	Subjects	Administration route	Dose (/kg)	Amnesic effect obtained?
Protein synthesis inhibitors					
DNA and protein synthesis interference	Anisomycin	Mice	i.p.	75 mg	+
		Mice	i.p.	150 mg	+ and +/−^1, 3^
		Mice	i.p.	225 mg	+/−
		Mice	s.c.	50 mg	*
		Rats	i.p.	50 mg	+
mRNA translocation interference	Cycloheximide	Rats	i.p.	2.2 mg	+
Receptor antagonists					
Oxytocin receptor	Atosiban	Rats	i.p.	0.001–1 mg	-
Adenosine receptor	Caffeine	Rats	i.p.	20 mg	+/−^1^
GABA_A_-R (partial agonist)	Flumazenil	Rats	i.p.	1 mg	+
NMDA-R	MK-801	Mice	i.p.	0.03 mg	+
		Mice	i.p.	0.06 mg	+
		Mice	i.p.	0.1 mg	+ and +/−
		Mice	i.p.	0.12 mg	+
		Rats	i.p.	0.1 mg	+ and +/−^3^ and -
Opioid receptor	Naloxone	Rats	i.p.	3 mg	+/−^3^
β-Adrenergic receptor	Propranolol	Mice	i.p.	10 mg	- and +/−
		Rats	i.p.	2 mg	-
		Rats	i.p.	5 mg	+ and -
		Rats	i.p.	10 mg	+ and -
Dopamine D1/D5 receptor	SCH23390	Rats	i.p.	0.1 mg	-
CB1-R (partial agonist)	SR141716A	Mice	i.p.	1–10 mg	-
Histamine H3-R (inverse agonist)	Thioperamide	Mice	i.p.	2.5–30 mg	-
	Pitolisant	Mice	i.p.	1.25–20 mg	-
Receptor agonists					
GABA_A_-R	Betulin (BE)	Rats	oral	2 mg	-
	Betulinic Acid (BA)	Rats	oral	2 mg	-
	BE + BA	Rats	oral	2 mg	+
	Ethanol	Rats	i.p.	0.5/1/1.5 mg	+/−^4^
	*Souroubea sympetala*	Rats	oral	8/25/75 mg	+/−^4^*
α_2_-Adrenergic receptor	Clonidine	Mice	i.p.	0.3 mg	+/−^1^
		Rats	i.p.	0.1 mg	-
		Rats	i.p.	0.3 mg	+
μ-Opioid receptor	Morphine	Rats	s.c.	7.5 mg	*
NOP receptor	Ro 65–6570	Mice	i.p.	0.1/1 mg	+/−^4^
	AT-403	Mice	i.p.	0.03/0.1 mg	+/−^4^
Benzodiazepines					
GABA_A_ receptor agonist	Diazepam	Rats	oral	1/2 mg	+
GABA_A_ receptor (allosteric modulator)	Midazolam	Rats	s.c.	1 mg	+
		Rats	i.p.	1 mg	+ and -
		Rats	i.p.	1.5 mg	+ and - and +/−^1, 2, 3^
		Rats	i.p.	1/1.5/3 mg	+/−^4^
		Rats	i.p.	2 mg	+
		Rats	i.p.	3 mg	+ and - and +/−^3^
Cannabinoids					
Indirect potentiation of CB1-R-mediated transmission	CBD	Rats	i.p.	1 mg	+/−
		Rats	i.p.	3 mg	+
		Rats	i.p.	10 mg	+/−
		Rats	i.p.	30 mg	+
		Rats	oral	50 mg	+
Activation of cannabinoid system	Cannabis plant extracts (after isolation of THC and CBD)	Rats	oral	43 mg	+
CB1-R agonist	THC	Rats	oral	5 mg	-
		Rats	i.p.	0.1/0.3/1/10 mg	+/−^4^
Intracellular molecule inhibitors					
DNA ligases and polymerases	Ara-C	Mice	i.p.	1000 mg	-
GSK-3	AR-A014418	Mice	i.p.	30 mg	+
NF-κB	DDTC	Rats	i.p.	200 mg	+
11β-hydroxylase	Metyrapone	Rats	i.p.	75 mg	-
PARP-1	Tiq-A	Mice	i.p.	0.5 mg	+
Other					
Hormone	Corticosterone	Rats	i.p.	1/3/10 mg	+/−^1, 4^
Peptide	GRP	Rats	i.p.	10 nmol	+
Bacterial toxin	Lipopolysaccharides	Mice	i.p.	125 μg	+
NE-DA reuptake inhibitor	Methylphenidate	Rats	i.p.	3/10 mg	-
DA reuptake inhibitor	Modafinil	Mice	i.p.	200 mg	+
AMPA receptor potentiator	PEPA	Mice	i.p.	30 mg	-
Glutamatergic system blocker	Riluzole	Rats	s.c.	0.1/0.3/1/3 mg	+/−^4^

Additional details for each study, including PubMed ID, strain, duration of the reactivation session (ranging from 30 s to 10 min), time of drug administration, and time between training and reactivation session (ranging from 1 to 36 d), are available at https://osf.io/x2pkq/. Ara-C = 1-β-D-arabinofuranosylcytosine triphosphate; CBD = cannabidiol; DA = dopamine; DDTC = diethyldithiocarbamate; GRP = gastrin releasing peptide; NE = norepinephrine; PEPA = 4-[2-(phenylsulfonylamino)ethylthio]−2,6-difluorophenoxyacetamide; s.c. = subcutaneous; THC = Δ9-tetrahydro-cannabinol; + = at least one study reported a statistically significant amnestic effect; * = amnestic effect was found to be transient; - = at least one study reported a non-significant effect; +/− = at least one study observed that the amnestic effect occurred under some conditions:

1 depending on training parameters (e.g., shock intensity).

2 depending on memory age.

3 depending on reactivation duration.

4 depending on drug dose.

**Table 4:** Overview of studies in which pharmacological agents were administered *intracranially* with the aim of inducing reactivation-dependent amnesia for contextual fear memories in rodents, as identified by the systematic review

Target/function	Drug	Subjects	Administration route	Dose	Amnesic effect obtained?
Protein synthesis inhibitors					
DNA and protein synthesis interference	Anisomycin	Mice	ACC	50 μg	-
		Mice	BLA	62.5 μg/side	+
		Mice	CA1	60 μg/side	+/−^3^
		Mice	CA1	62.5 μg/side	+ and +/−^2, 3^
		Mice	dHipp	62.5 μg/side	+
		Mice	dHipp	75 μg	+/−^2^
		Mice	mPFC	62.5 μg/side	-
		Mice	i.c.v.	0.1 mg	+/−^3^
		Rats	ACC	62.5 μg/side	+
		Rats	BLA	62.5 μg/side	+
		Rats	CA1	80 μg/side	*
		Rats	CA1	250 μg/side	+
		Rats	MC	62.5 μg/side	-
RNA Polymerase II	DRB	Rats	CA1	10 ng/side	+
Receptor antagonists					
CB1-R (inverse agonist)	AM251	Rats	amygdala	280 pg	+ and -
NMDA-R	D-AP5	Rats	dHipp	5 μg/side	+
β-Adrenergic receptor	Propranolol	Rats	BLA	1.25 μg/side	+
5-HT6-R	SB-271046	Rats	CA1	10 μg/side	-
5-HT5A-R	SB_6_99551	Rats	CA1	10 μg/side	+/−
mAch-R	Scopolamine	Rats	amygdala	50 μg	-
Histamine H3-R (inverse agonist)	Thioperamide	Rats	amygdala	44 pg	-
Receptor agonists					
GABAa-R	Muscimol	Rats	IL	4 nmol/side	-
		Rats	PL	4 nmol/side	+
5-HT7-R	AS-19	Rats	CA1	5 μg/side	-
5-HT6-R	WAY-208466	Rats	CA1	0.04 μg/side	+/−
Cannabinoids					
CB1 and CB2-R agonist	Anandamide	Rats	CA1	0.17 ng/side	+
CP55,940		Rats	CA1	2.5 μg/side	+
		Rats	IL	2.5 μg/side	+
		Rats	RSC	2.5 μg/side	+
Intracellular molecule inhibitors					
PARP-1	3-aminobenzamide	Mice	dHipp	18 μg/side	+
		Mice	mPFC	18 μg/side	-
	PJ34	Mice	dHipp	0.2 mM/side	+
LIM kinase	BMS-5	Rats	CA1	200 μm/side	+
PKC	Chelerythrine	Rats	PL	3 nmol/side	+/−
PKMζ	ZIP	Rats	PL	10 nmol/side	+/−
MEK	U0126	Rats	dHipp	2/4 μg/side	-
IKK	Sulfasalazine	Rats	dHipp	2 μg/side	+ and -
		Rats	i.c.v.	5/10 mM	+/−^4^
Proteasome	β-lac	Rats	dHipp	32 ng/side	-
Calpain	ALLN	Mice	CA1	1 μg/side	+
	PD150606	Rats	CA1	0.153 ng/side	+
Rac	NSC23766	Rats	BLA	5 μg/side	-
		Rats	CeA	5 μg/side	-
		Rats	CA1	5 μg/side	+
mTOR	Rapamycin	Rats	dHipp	5 μg/side	+
N-glycosylation inhibition	Swainsonine	Mice	dHipp	0.5 μg/side	+
	1-deoxynojirimycin	Mice	dHipp	16 μg/side	+
	Tunicamycin	Mice	dHipp	0.5 μg/side	+
Other					
Glutamatergic system blocker	Riluzole	Rats	dHipp	2 μm/side	+
Sodium channel blocker	Tetrodotoxin	Rats	Ent	5 ng/side	+
		Rats	amygdala	5 ng/side	+
Hormone	Angiotensin II	Rats	CA1	0.5 nmol/side	*
Peptide	Nociceptin	Mice	i.c.v	1/3 nmol	+/−^4^
Cytokine	IL-1β	Rats	CA1	5 ng/side	+

Additional details for each study, including PubMed ID, strain, duration of the reactivation session (ranging from 1 to 10 min), time of drug administration, and time between training and reactivation session (ranging from 1 to 36 d), are available at https://osf.io/x2pkq/. ACC = anterior cingulate cortex; ALLN = N-Acetyl-Leu-Leu-norleucinal; BLA = basolateral amygdala; CeA = Central amygdala; dHipp = dorsal hippocampus; D-AP5 = D-2-amino-5-phosphonovaleric acid; DRB = 5,6-dichloro-1-b-dribofuranosylbenzimidazole; Ent = entorhinal cortex; IL = infralimbic cortex; i.c.v. = intracerebroventricular; MC = motor cortex; mPFC = medial prefrontal cortex; PL = prelimbic cortex; RSC = retrosplenial cortex; + = at least one study reported a statistically significant amnestic effect; * = amnestic effect was found to be transient; - = at least one study reported a non-significant effect; +/− = at least one study observed that the amnestic effect occurred under some conditions (superscripts see Table 3).

**Figure 3.**
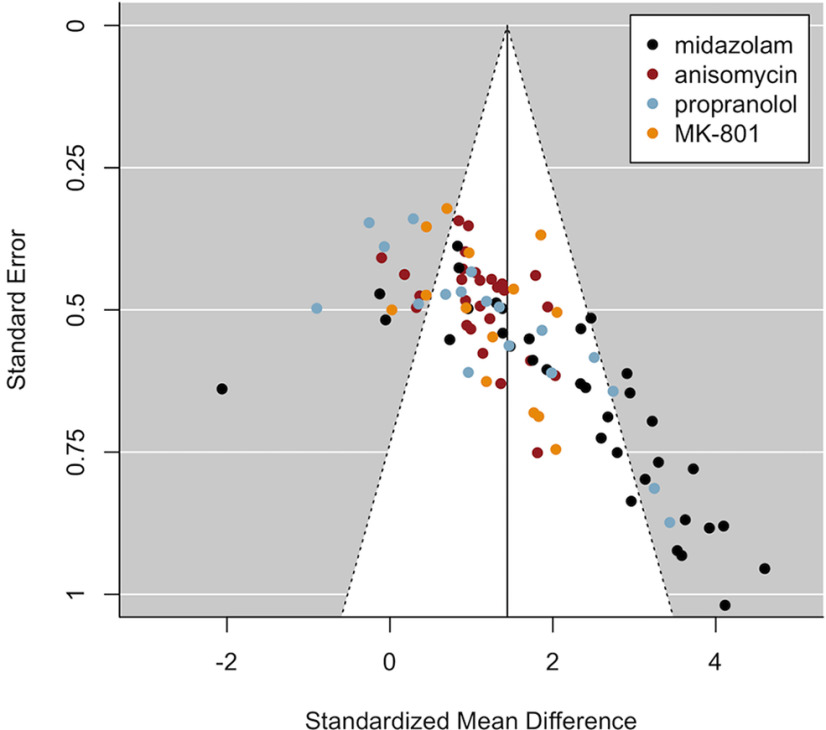
Funnel plot including published studies suggests biased effect sizes. The asymmetrical funnel shape observed here was statistically confirmed by Egger’s regression (*p* < 0.001) and is suggestive of biased study outcomes because of selection of significant results for publication.

**Figure 4.**
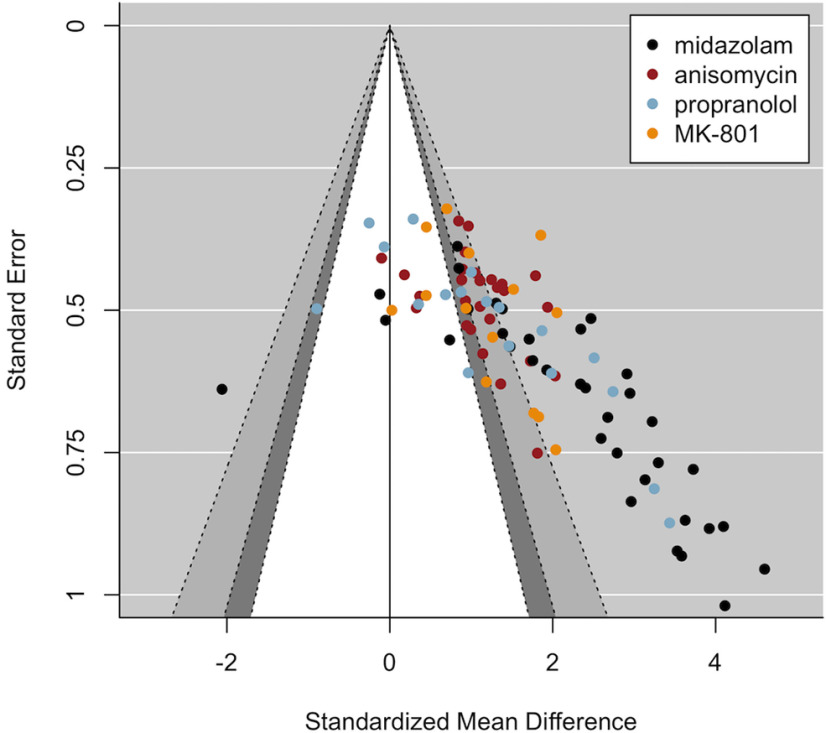
Contour-enhanced funnel plot including published studies suggests publication bias. The white area and the region on its left side contain studies with statistically non-significant amnestic effects based on one-tailed tests (drug < control; *p* ≥ 0.05). The plot suggests that non-significant studies are missing in the literature (i.e., publication bias). Remarkably, effect sizes are most densely plotted at the border of statistical significance, which might also imply biased effect sizes.

The random-effects meta-analysis on this sample (k = 95, *N* = 1896) showed heterogeneity in effect estimates between studies [i.e., variation in effect estimates beyond chance; Q(94) = 334.08; *p* < 0.001; τ^2^ = 0.80 (SE = 0.16) [0.61; 1.43]; I^2^ = 75.26% [69.93; 84.44]]. Because of this statistical heterogeneity, and as preregistered, amnestic drug and research group were included as moderators, and were found to be significant [QM(33) = 68.64, *p* < 0.001]. Nevertheless, residual heterogeneity (after considering those moderators) remained significant [QE(61) = 158.14; *p* < 0.001; τ^2^ = 0.53 (SE = 0.15) [0.32; 1.07]; I^2^ = 64.97% [53.02; 79.12]]. I^2^, the percentage of the variability in effect estimates that is because of heterogeneity rather than chance, decreased from 75% (considerable) to 65% (substantial) after inclusion of the moderators (Deeks et al., 2019). The funnel plot that includes all 95 drug-vehicle comparisons suggests asymmetry (Fig. 3), which was confirmed statistically by Egger’s test (*t*_(60)_ = 5.04, *p* < 0.001 for the model including drug and research group as moderators). As mentioned before, one way to distinguish publication bias from other sources of asymmetry is by adding contours of statistical significance to the funnel plot. Such a contour-enhanced funnel including all published drug-vehicle comparisons (Fig. 4) illustrates that studies are missing in the area of statistical non-significance, adding credibility to publication bias being a source of asymmetry. In addition, effect sizes are most densely plotted in the gray area at the border of statistical significance, which might suggest a biased distribution of effect sizes (Simonsohn et al., 2014). Overall, the results based on all selected published studies (using ANI, MDZ, PROP, or MK-801) are in line with those from our pilot study (which only included MDZ), as evidence for publication bias was observed in both sets of analyses.

The majority of the included published drug-vehicle comparisons (i.e., 82%) was reported as statistically significant, and over 90% of the published papers concluded that amnesia could be obtained under at least some of the applied standard conditions (i.e., conditions in which the amnestic effect is expected to occur based on theoretical considerations; see Inclusion criteria). From the 12 published papers reporting non-significant amnestic effects under standard conditions, eight papers did find amnesia in some of the applied standard conditions (i.e., when changing the duration of the reactivation session, when administering ANI instead of PROP, or when infusing the amnestic drug into a different brain area), which leaves a total of four papers (including six comparisons) that found no amnestic effect under standard conditions whatsoever. Most of them did, however, obtain amnesia when using multiple injections (albeit temporarily; Lattal and Abel, 2004), when using a knock-out mice model (Yamada et al., 2009), or when postreactivation MK-801 injection was preceded by prereactivation injection of the cannabinoid CB1 receptor agonist arachidonyl-2-chloroethylamide (ACEA; Lee and Flavell, 2014). Only one of the included papers reported an overall failure to induce amnesia (using PROP; Careaga et al., 2015).

### Funnel plots and Egger’s regression suggest publication bias when excluding MDZ studies

Below, we report the results of additional analyses that were not part of the preregistered analysis plan, but that allow for a clearer interpretation of the current findings. Visual inspection of the funnel plot including all studies (Figs. 3, 4) seems to suggest that MDZ studies (plotted in black) strongly contribute to the asymmetrical funnel shape, or, in other words, to the observed correlation between the effect sizes and their SEs suggestive of publication bias. Therefore, we exploratorily repeated the analyses excluding the MDZ studies, to assess whether the same conclusions would still hold when solely looking at the three other amnestic drugs. In addition, we repeated the analyses for each drug separately.

**Figure 5.**
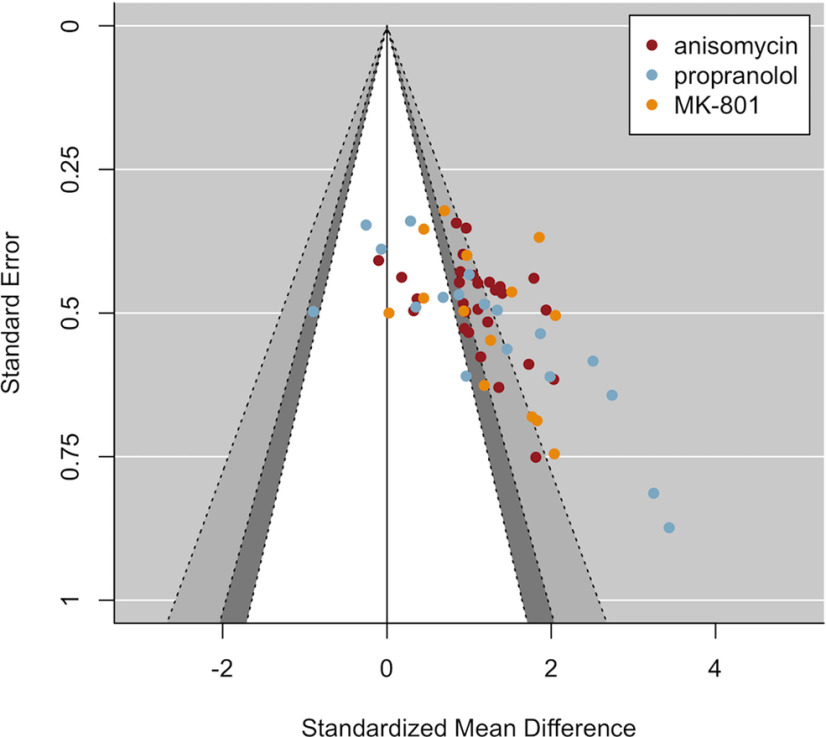
Contour-enhanced funnel plot still suggests publication bias when MDZ studies are excluded. The white area and the area on its left side contain studies with statistically non-significant amnestic effects based on one-tailed tests (drug < control; *p* ≥ 0.05). The asymmetrical funnel shape observed here was statistically confirmed by Egger’s regression (*p* < 0.001; or with Research Group and Drug as moderators: *p* = 0.022; exploratory analysis) and is suggestive of biased study outcomes because of selection of significant results for publication.

The plot with ANI, PROP, and MK-801 studies (i.e., excluding MDZ; Fig. 5) still showed an asymmetrical funnel shape, which was confirmed statistically by Egger’s regression, even when taking into account the moderating influence of drug and research group (*t*_(32)_ = 2.41, *p* = 0.022), albeit to a lesser extent compared with when MDZ was included. When inspecting the results for each drug individually [see “5. Funnel plots per Drug (exploratory analyses)” at https://osf.io/zshwx/], asymmetrical funnel shapes were observed for MDZ, ANI and PROP, but not for MK-801. In addition, asymmetry was no longer observed for ANI (*t*_(12)_ = 0.95, *p* = 0.362) or PROP (*t*_(10)_ = 1.04, *p* = 0.321) when including research group as a moderator, implying evidence for overall asymmetry for ANI and PROP, but no evidence for asymmetry within research groups.

### Unpublished data contain proportionally more failures to replicate than published data

**Figure 6.**
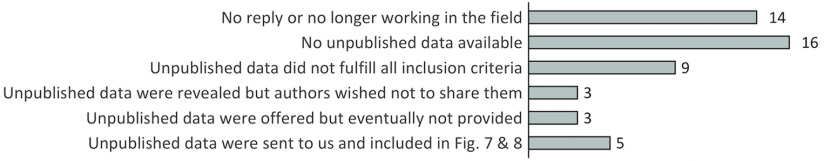
Replies to our request for unpublished data (clustered per research group).

We contacted all corresponding authors from the research articles that were included in the meta-analysis and sent out an E-mail to the Pavlovian Society mailing list to enquire about and request unpublished datasets. In addition, a request for unpublished data were posted on StudySwap (https://osf.io/98dr6/wiki/home/) and ResearchGate (https://bit.ly/34xllde). Figure 6 provides an overview of the received responses.

Most researchers did not reply to our emails or replied that they did not have any unpublished data available (note that those two “replies” together comprised 60% of the responses). Some researchers provided information about unpublished studies that did not meet all our inclusion criteria. For example, three researchers replied having unpublished reconsolidation data from studies using cued fear memories. There were also three cases in which contextual fear conditioning was used, but the reactivation session was too long for inclusion (i.e., longer than two times the duration of the training session). For two of these, the outcome was also shared, one in which amnestic effects of PROP and MDZ were found and another one in which no effect of PROP was found. In another series of studies, an excluded amnestic agent, i.e., cycloheximide, was used. A dose of cycloheximide that was found to affect retention when injected after conditioning did not induce amnesia when given after a memory reactivation session despite varying the parameters of training (0.5- or 0.7-mA shocks) and reactivation (3 or 5 min) in a series of four studies described in an undergraduate student’s report (Zacouteguy Boos et al., 2013). Researchers from three different research groups reported to have an (extensive) series of unpublished studies meeting all our inclusion criteria but wished not to share the data for inclusion in the current analyses. Finally, three researchers (from three different research groups) offered to share their data but did not manage to timely access and/or send those data. We did receive unpublished data that could be included in the current meta-analyses from seven researchers from five different research groups (a total of 12 drug-vehicle comparisons). Importantly, the amount of unpublished data that we could include in the current manuscript is less than half of all the unpublished data disclosed to exist to us by the contacted researchers. Overall, it appears that statistically non-significant results from reconsolidation studies in rodents are less likely to be published and, in some cases, researchers were unable or reluctant to share such “negative” data for the current paper.

**Figure 7.**
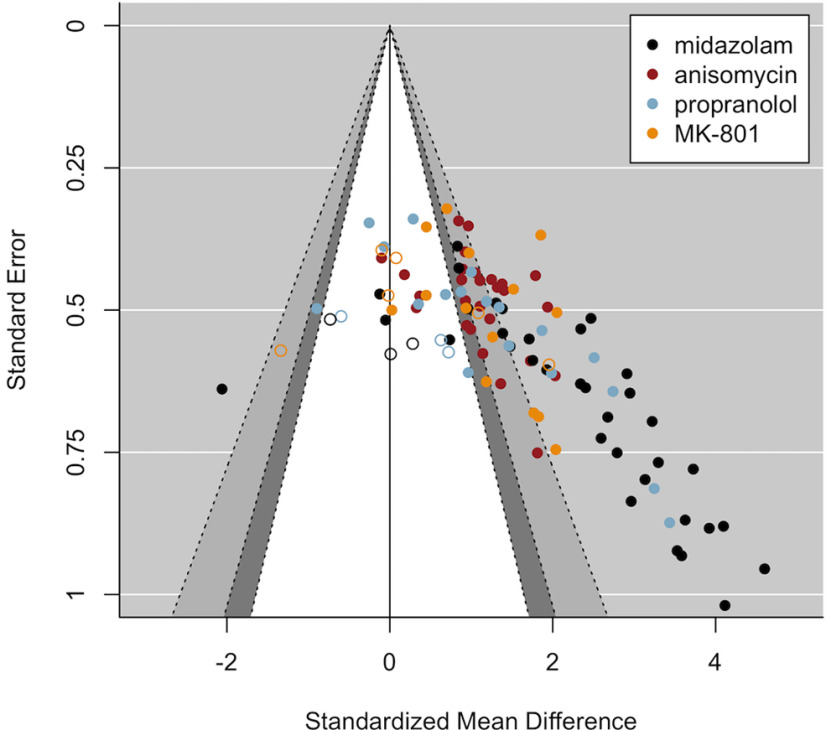
Contour-enhanced funnel plot of published (filled circles) and unpublished (empty circles) studies. In contrast to published results (95 drug-vehicle comparisons), studies that remained unpublished (12 drug-vehicle comparisons) showed smaller, and mostly non-significant, amnestic effects. This discrepancy between published and unpublished results is in line with the presence of publication bias that was suggested by the funnel plots. The majority of unpublished “negative” studies of which the existence was revealed could not be included in the current study because of author preferences.

**Figure 8.**
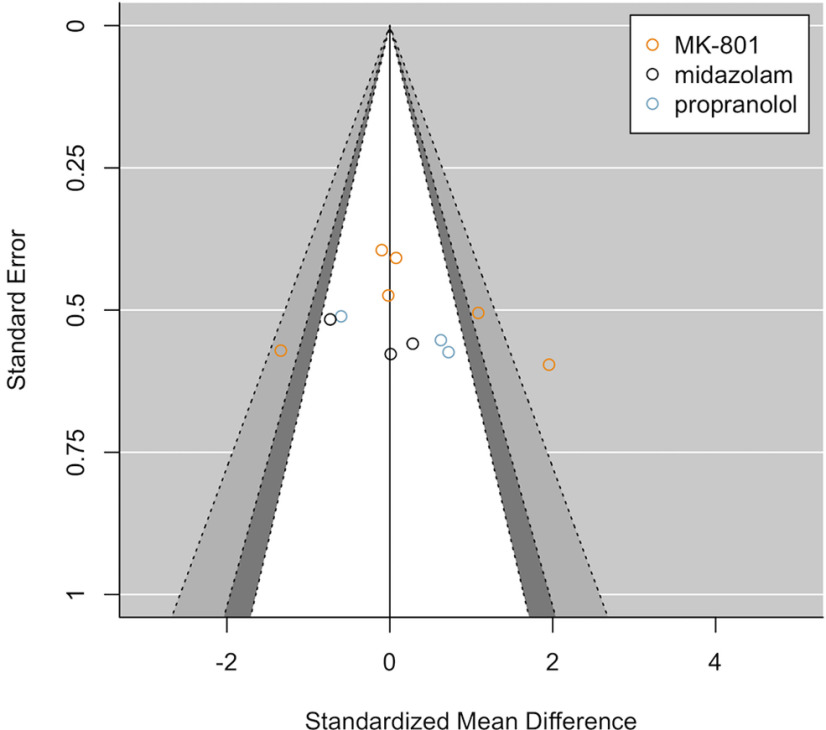
Contour-enhanced funnel plot of unpublished studies. A relatively symmetrical pattern is observed and, in line with this observation, Egger’s regression suggests that there is no statistically significant evidence for asymmetry (*t*_(10)_ = 0.70, *p* = 0.499).

The obtained unpublished studies that met all our inclusion criteria are plotted in combination with the published data (Fig. 7) and alone (Fig. 8). A total of 12 drug-vehicle comparisons was included, in which either MK-801 (six studies), PROP (three studies), or MDZ (three studies) was administered before or after a contextual fear memory reactivation session. One (MK-801) study contained two intervention groups that were compared with the same control group, so the intervention groups were combined into a single group as recommended by Higgins and Green (2011). A detailed overview of the adopted parameters of those studies can be found at https://osf.io/gfwrj/. The funnel plot including all studies (Fig. 7) still shows asymmetry after inclusion of the obtained unpublished data (*t*_(70)_ = 5.63, *p* < 0.001; with drug and research group as moderators). This was not unexpected given that we probably did not track down all existing unpublished data and because a large part of the unpublished data that we did uncover were eventually not shared by the authors for inclusion in the current paper. Importantly, studies that previously remained unpublished show smaller and mostly statistically insignificant effect sizes compared with those reported in the literature (Fig. 7). Although the limited amount of unpublished data does not allow for robust conclusions, the symmetrical funnel shape that is observed when plotting unpublished datasets only (Fig. 8) suggests no evidence for bias in our sample of unpublished studies (*t*_(5)_ = –0.34, *p* = 0.751). Only 20% of the shared unpublished experiments found a statistically significant amnestic effect (significance based on one-sided *t* tests), while around 80% of the published experiments reported statistically significant amnestic effects. An exploratory Fisher’s exact probability test suggested that those proportions between published and unpublished studies were significantly different (*p* < 0.001). Note that any conclusions drawn from such a comparison should be interpreted with caution, given that the obtained unpublished data may not be representative of all existing unpublished data. An exploratory (i.e., not preregistered) random-effects meta-analysis with publication status (i.e., whether a study was published or unpublished) as a moderator revealed that publication status significantly moderated the size of amnestic effects [QM(1) = 17.06, *p* < 0.001].

## Discussion

Our own extensive experience with drug-induced, reactivation-dependent amnesia for contextual fear memories in rats (Schroyens et al., 2019a,b) suggested that amnestic effects are not easily found, even when performing well-powered, exact replication attempts of published “positive” studies and trying out a wide variety of experimental parameters in several different laboratories. Of note, we also failed to obtain amnestic effects using behavioral or pharmacological interventions for cued fear memories in rats (Luyten and Beckers, 2017; Luyten et al., 2020) or healthy human participants (Schroyens et al., 2017; Chalkia et al., 2019, 2020a). Those observations, although corroborated by personal communication with experts in the field, were in stark contrast with the published literature, which contains a plethora of significant (mostly large) amnestic effects and hardly any negative results. This discrepancy inspired us to formally investigate publication bias.

We performed a systematic PubMed search and selected studies that aimed to induce reactivation-dependent amnesia for contextual fear memories in rodents under standard conditions with a commonly-used amnestic drug (i.e., ANI, MDZ, PROP, or MK-801; see above, Inclusion criteria). The majority of the 95 included published drug-vehicle comparisons (i.e., 80%) was reported as statistically significant and funnel plots and Egger’s linear regression provided evidence for publication bias in this sample. Only one of the included papers reported an overall failure to induce amnesia (Careaga et al., 2015). In contrast, the data that we received from previously unpublished studies mostly consisted of “negative” findings, as around 80% did not find a statistically significant amnestic effect. This discrepancy between published and unpublished results further supports the presence of publication bias. It should be mentioned that part of the unpublished experiments that we were informed of could not be included in the current study due the inability or reluctance of some of researchers to share relevant information about their unpublished findings. In any case, the current results suggest that the literature on reactivation-dependent amnesia for contextual fear memories in rodents is biased.

Possible sources of publication bias can be found at different stages, such as author submission, peer review or editorial decisions (in which the journal’s policy may play a role; Song et al., 2009). Authors’ decisions not to submit their negative results for publication can result from (1) the fact that such results are considered unimportant, (2) fear of debunking own previously-published results, theories or conclusions, or (3) the expectation of rejection by (prestigious) journals. Importantly, in the presence of publication bias, the published studies as a whole do not provide solid evidence concerning the reliability of reactivation-dependent amnesia. Selective publication of research findings depending on their statistical outcome results in the literature painting an overly optimistic picture, with misleading overall estimates of the size and replicability of amnestic effects. This false image, in turn, may result in researchers investing time and resources on an effect that seemed to be robust but may turn out to be non-replicable or, at least, difficult to replicate.

Based on the evidence for publication bias provided here and the results of our empirical studies in which no evidence for reactivation-dependent amnesia was obtained (Schroyens et al., 2019a,b), we do not claim that such phenomenon for contextual fear memories in rodents does not exist, nor do we intend to doubt the veracity of the published studies included here; but we do conclude that drug-induced reactivation-dependent amnesia for contextual fear memories in rodents is far less robust than what is projected by the existing literature. In light of other empirical studies from our and other labs that reported failures to replicate, the same may apply to cued fear memories in rodents (Luyten and Beckers, 2017; Luyten et al., 2020) and healthy humans (Bos et al., 2014; Thome et al., 2016; Schroyens et al., 2017; Chalkia et al., 2019, 2020a). We want to point out that the intuitive reasoning of an effect being truly existent based on it being reported many times can be problematic, as it has been suggested that such counting ignores reporting bias, selection bias and questionable research practices (Hedges and Olkin, 1985; Vadillo et al., 2016). Likewise, non-significant results should be interpreted as the absence of evidence for rather than the evidence of absence of a treatment effect (Taleb, 2007) and the observation of a statistically non-significant result should not be equalized with the underlying theory being wrong (Meehl, 1990).

It is good to note that publication bias is probably by no means unique to the reconsolidation field; it is likely to hinder accurate estimation of effect sizes for many other (behavioral) phenomena as well. In this paper, we focused on a delineated part of the reconsolidation literature to systematically investigate publication bias, allowing us to illustrate the existence and pervasiveness of publication bias in this particular research domain. The obtained results provide us with a clearer view on the potential translational value of reactivation-dependent amnesia for fear memories. We strongly believe that other research areas may also benefit from systematic investigations that (dis)confirm (1) the existence of publication bias and, if applicable, (2) shed light on its extent.

It should be noted that publication bias is only part of the story. Our own failures to exactly replicate prior “positive” studies already suggested that study outcome could depend on the lab in which the study was performed, or at least, that the outcome depends on subtle and unknown factors that differ between labs. In line with our experiences, the current meta-analysis suggested that the size of the amnestic effect depends on the research group in which the experiment was performed. Nevertheless, also within research group and amnestic drug, statistically significant between-study heterogeneity was observed, suggesting that observed effect sizes show differences beyond those to be expected by chance. Such heterogeneity indicates that the size of the amnestic effect, even under standard conditions, is expected to vary significantly. In combination with the current evidence for publication bias and the range of identified null findings, this implies that the outcome of postreactivation amnestic treatments is unpredictable. In addition, dominant theories (e.g., reconsolidation, state dependency) in their current form are unable to pinpoint which factors exactly influence the occurrence and size of reactivation-dependent amnestic effects. The need to define moderators of amnestic effects that has often been mentioned in reply to replication failures might be interesting for further development or refinement of theories on reactivation-dependent amnesia provided that data-driven moderators are also empirically tested.

The lack of robustness of reactivation-dependent amnesia, in combination with the strict (but vague) conditions that are required for memory destabilization and the absence of clear explanations for some of the observed null effects, cast doubt on the potential of the proposed clinical application of postreactivation interventions for the treatment of phobias or posttraumatic stress disorder (PTSD). Indeed, studies in (sub)clinical samples have not been entirely convincing (Brunet et al., 2011; Wood et al., 2015; Kindt and van Emmerik, 2016; Elsey et al., 2020; for an overview, see Beckers and Kindt, 2017). Importantly, the mixed results obtained in clinical studies might not reflect issues with translation from basic to clinical science but may simply reflect the lack of robustness of results obtained in basic research and illustrate the lack of insight in the optimal and boundary conditions for reactivation-dependent memory interference.

For the field to evolve, replication and non-biased publication of obtained results is essential. The classical publication system clearly favors the publication of novel or “positive” results, but there is a set of valuable new tools that create opportunities to increase transparency, reproducibility and credibility of research findings. For example, documentation of hypotheses, research design, and/or planned analyses on a public repository before commencing data collection, referred to as “preregistration,” ensures a clear distinction between hypotheses and/or analysis plans that were formulated before versus after observing the results and can be made publicly accessible on paper publication (Nosek et al., 2018). The OSF is an online platform that can be used for such preregistration and for the sharing of data, analyses scripts, etc. (http://osf.io). Making datasets and analysis scripts publicly available provides the opportunity to be transparent and enhance credibility of one’s obtained results and conclusions (Klein et al., 2018). Nevertheless, while valuable, those tools mostly provide a means to an end, as verification of agreement between registered and performed analyses must be assured and analytic reproducibility of published results needs to be checked. One valuable publication format in this regard is the Verification Report, in which authors of an empirical article reanalyze the original study data using the reported analyses to verify whether the same conclusion can be drawn as those reported in the original article (Chambers, 2020; see Chalkia et al., 2020b for an example from the reconsolidation field). Finally, the use of Registered Reports, in which in-principle acceptance for publication is granted before data collection for a study commences, assures inclusion of study results in the published record on the basis of quality of the methods, regardless of a study’s outcome (Hardwicke and Ioannidis, 2018). This format removes the pressure to come up with statistically significant findings for publication and prevents publication bias (https://cos.io/rr provides helpful guidelines for the submission of a Registered Report and an extensive list of participating journals). Researchers can thus take the opportunity of using those tools to increase transparency and reproducibility, both of which are essential for the reconsolidation field (and empirical science in general) to move forward.

## Synthesis

Reviewing Editor: Laura Bradfield, University of Technology Sydney Decisions are customarily a result of the Reviewing Editor and the peer reviewers coming together and discussing their recommendations until a consensus is reached. When revisions are invited, a fact-based synthesis statement explaining their decision and outlining what is needed to prepare a revision will be listed below. The following reviewer(s) agreed to reveal their identity: NONE.

Thank you for your additions, changes, and revisions, this manuscript is now in great shape. Apologies for the delay in making this decision - one of the original reviewers was unable to re-review it so I wanted to make sure I had a chance to look over it myself in some detail, which I have now had a chance to do. The one reviewer that did re-review found all the issues adequately addressed and had no further comments.

As for myself, I had no major issues that need addressing, but I do have a few minor ones.

• Lines 264-265 - “was found to be a significant moderator...” moderator of the heterogeneity? The position on the funnel plot? If this could be clarified in text it would help me understand it.

• Line 272-273 - “there was a significant positive relationship between the effect estimates and their standard errors” - please clarify what this indicates in text.

• Line 384. I wonder if having the word ‘reminder‘ in parentheses is necessary? If not please remove it otherwise it comes across a little accusatory.

• Likewise for lines 403-404, starting with and, in some cases...”. Does this need to be said? I’m open to an argument for its inclusion, I just wonder if it comes across as implying that the researchers did not want a paper published suggesting that the effect was not robust. Unless we explicitly know that this is the case (because they said so) then I think it’s best to leave out this passage. There could be any number of reasons why a researcher may not share unpublished results (maybe a grad student made a mistake? Maybe a fire alarm went off in the middle of the experiment - both things that have happened to me!). I will note that where this is listed as a possibile reason for why researchers did not share their data in the discussion is fine and doesn’t seem accusatory in the same manner.

• I think I mentioned this on the review of the first stage registered report, but I again have the concern that should we take any behavioural effect and ask people for their unpublished data, we are going to find evidence of publication bias and a less than robust effect that what appears from published data. Data that remains unpublished is, by its nature, more likely to be non-significant. I remember that the authors provided some rebuttal to this point, but I would still like some kind of acknowledgement in the discussion that future studies might reveal a similar lack of robustness for other behavioural effects should a similar process to that undertaken here be applied to it. Off the top of my head, I know I had a hard time getting overexpectation to work, so I wonder if that is also a not-very-robust effect that has benefitted from publication bias. However, the authors need not be that specific - please just include a sentence somewhere in the discussion mentioning the possibility of reactivation-dependent amnesia for contextual fear memories not being the only possible effect suffering from such bias.

## Author Response

Dear Dr. Bradfield,

Thank you for going through our Stage 2 manuscript in detail and for your comments and suggestions. We address your comments below, and changed the manuscript accordingly (marked in red font).

- Lines 264-265 - “was found to be a significant moderator...” moderator of the heterogeneity? The position on the funnel plot? If this could be clarified in text it would help me understand it.

We added the following information to the manuscript:

"Given such heterogeneity, and as preregistered, research group was included in the model as a moderator. We found that the effect sizes plotted in Fig. 1 (left panel) depended on the research group in which the experiment was performed (i.e., research group was a statistically significant moderator of effect size; QM(5) = 82.35, p < .001).”

- Line 272-273 - “there was a significant positive relationship between the effect estimates and their standard errors” - please clarify what this indicates in text.

We added the following information to the manuscript:

"Doing so, Egger’s test provided statistical evidence for funnel plot asymmetry (t(27) = 5.02; p < .001), which can be an indication of publication bias.”

- Line 384. I wonder if having the word ‘reminder’ in parentheses is necessary? If not please remove it otherwise it comes across a little accusatory.

We removed ‘reminder’ from the text.

- Likewise for lines 403-404, starting with and, in some cases...”. Does this need to be said? I’m open to an argument for its inclusion, I just wonder if it comes across as implying that the researchers did not want a paper published suggesting that the effect was not robust. Unless we explicitly know that this is the case (because they said so) then I think it’s best to leave out passages such as this. There could be any number of reasons why a researcher may not share unpublished results (maybe a grad student made a mistake? Maybe a fire alarm went off in the middle of the experiment - both things that have happened to me!).

We had written that “...in some cases, researchers were unable or reluctant to share such ‘negative’ data for the current paper”. In addition, in the Results section we had pointed out that “Researchers from three different research groups reported to have an (extensive) series of unpublished studies meeting all our inclusion criteria but wished not to share the data for inclusion in the current analyses” (lines 388-390).

None of those researchers explicitly said that they did not want their results to be included in our meta-analysis due to the fact that those studies illustrated that the effect was not robust. Below, we would like to elaborate on the communication that we had with those researchers that led us to include the sentences above in the manuscript. We hope that you agree that there are sufficient arguments for including this in the manuscript, but if not, please let us know.

The arguments were based on confidential conversations with the researchers involved and were removed from the published version of the rebuttal letter because the authors wished to keep this information private.

-I think I mentioned this on the review of the first stage registered report, but I again have the concern that should we take any behavioural effect and ask people for their unpublished data, we are going to find evidence of publication bias and a less than robust effect that what appears from published data. Data that remains unpublished is, by its nature, more likely to be non-significant. I remember that the authors provided some rebuttal to this point, but I would still like some kind of acknowledgement in the discussion that future studies might reveal a similar lack of robustness for other behavioural effects should a similar process to that undertaken here be applied to it. Off the top of my head, I know I had a hard time getting overexpectation to work, and it seems a highly parameter-specific effect from my experience, so I wonder if it is also a not-very-robust effect that has benefitted from publication bias (although the authors need not be that specific - please just include a sentence somewhere in the discussion mentioning the possibility of reactivation-dependent amnesia for contextual fear memories not being the only possible effect suffering from such bias).

We added the following paragraph to the manuscript (lines 474-481):

"It is good to note that publication bias is probably by no means unique to the reconsolidation field; it is likely to hinder accurate estimation of effect sizes for many other (behavioral) phenomena as well. In this paper, we focused on a delineated part of the reconsolidation literature to systematically investigate publication bias, allowing us to illustrate the existence and pervasiveness of publication bias in this particular research domain. The obtained results provide us with a clearer view on the potential translational value of reactivation-dependent amnesia for fear memories. We strongly believe that other research areas may also benefit from systematic investigations that (dis)confirm (1) the existence of publication bias and, if applicable, (2) shed light on its extent.”
